# 12. SYNAPTIC DYSFUNCTION IN SCHIZOPHRENIA: EXPLORATION OF NOVEL HYPOTHESES AND PROMISING NEW LEADS

**DOI:** 10.1093/schbul/sby014.043

**Published:** 2018-04-01

**Authors:** Laura Rowland

**Affiliations:** University of Maryland School of Medicine

## Abstract

**Overall Abstract:** Accumulating evidence suggests that bioenergetic function is impaired in the brain in schizophrenia. In normal brain, glucose is metabolized to lactate and pyruvate, which are monocarboxylate intermediates that serve as the primary energy source for neurons. Working memory and other cognitive domains are dependent on the shuttling of lactate from astrocytes to neurons. Defects in this complex pathway may underlie cognitive dysfunction in schizophrenia. The focus of this symposium is to present evidence of such defects, and to identify substrates that may be targeted for the development of new treatment strategies. Dr. Laura Rowland (University of Maryland, Baltimore, Maryland, USA) will present evidence of bioenergetic dysfunction in living subjects with schizophrenia. Increased levels of lactate (P < 0.05) were present in the ACC in schizophrenia (n = 27) compared to controls (n = 29). Higher lactate levels were associated with lowers scores on the MATRICS Consensus Cognitive Battery. These data establish a direct link between cognition and bioenergetic function in vivo in schizophrenia. Dr. Robert McCullumsmith (University of Cincinnati, Cincinnati, Ohio, USA) will present evidence of alterations in the lactate shuttle and glycolytic enzymes in postmortem samples from schizophrenia (n = 20) and control subjects (n = 20). Cell-subtype specific changes (P < 0.05) in transcripts include increased levels of the lactate transporter MCT4, decreased levels of the glycolytic enzymes PFK1 and hexokinase, and decreased levels of the glucose transporters Glut1 and Glut3. These data suggest attenuated glycolysis in pyramidal neurons, with a shift towards pathways that boost protection from oxidative stress. The last two speakers will present data that address mechanisms related to these findings, using animal models with behavioral endophenotypes of schizophrenia. Dr. Eduard Bentea (Free University of Brussels (VUB), Brussels, Belgium) will present data from the xCT knockout mouse showing that disruption of system xc-, which supports oxidative stress buffering mechanisms, leads to synaptic dysfunction. Specifically, electron microscopy studies indicate depletion of both pre-and post-synaptic glutamate, while electrophysiological studies show diminished excitatory postsynaptic potentials. These findings directly connect oxidative balance and extracellular glutamate levels with development of “broken” synapses, highlighting a potential mechanism for perturbation of bioenergetic coupling between astrocytes and neurons. Dr. Amy Ramsey (University of Toronto, Toronto, Canada) will present evidence from an animal model of synaptic dysfunction, the GluN1 knockdown mouse. These mice show a bioenergetic defect similar to schizophrenia, with decreased expression of glycolytic enzymes and glucose transporters. These translational findings indicate that genetic risk for schizophrenia may lead to an intermediate bioenergetic phenotype, where diminished supply of lactate and other energetic molecules to neurons could contribute to cognitive dysfunction. Taken together, the work presented by these speakers will provide a fresh look at the bioenergetic defects in schizophrenia, establishing that 1) metabolic perturbations in the brain are prominent and not just an effect antipsychotic treatment, 2) altered neuron-astrocyte coupling leads to synaptic dysfunction, and 3) genetic risk for “broken” synapses disrupts metabolic function.

